# Feature Review Papers on Gastroesophageal Junction and Gastric Cancers

**DOI:** 10.3390/cancers14163979

**Published:** 2022-08-17

**Authors:** Caroline Gronnier

**Affiliations:** 1 Eso-Gastric Surgery Unit, Department of Digestive Surgery, Magellan Center, Bordeaux University Hospital, 33600 Pessac, France; caroline.gronnier@chu-bordeaux.fr; Tel.: +33-(5)-5765-6005; Fax: +33-(5)-5765-6003; 2 Faculty of Medicine, Bordeaux Ségalen University, 33076 Bordeaux, France; 3 U1312 BRIC—BoRdeaux Institute in Oncology Inserm/Team 4 “Helicobacter-Associated Digestive Cancers, Cancer Stem Cells and Therapeutic Strategies”, 33076 Bordeaux, France

## 1. Introduction

Gastric cancer is the fifth most common cancer in the world and the third leading cause of cancer deaths [1]. This is due to a late diagnosis at the metastatic stage but also resistance to classical chemotherapies. Peri-operative chemotherapy is the reference treatment for resectable gastric and esophageal cancers. In recent years, research has been marked by the classification of gastric cancer based on molecular subtypes, which opens the possibility of personalized therapy. Targeted therapies have recently emerged that focus on microsatellite instability, human epidermal growth factor receptor 2, programmed cell death ligand 1 and Epstein–Barr virus. Only a small proportion of patients with these cancers are eligible for targeted therapies. In addition, many new signaling pathways involved in gastric cancer have been identified in recent years and the esogastric junction constitutes a potential lead for new target therapies. Research is particularly active in the field of immunotherapy and the targeting of cancer stem cells.

The objective of this Special Issue was to collect journal articles that provide the latest news on gastric and esogastric cancer.

## 2. Pathogenesis and Risks Factors

The identified biological differences between gastric cancers in Western and Eastern countries make the external validity of international randomized trials problematic. Indeed, gastric cancers in Western countries have more often signet ring cells and a proximal location than in Eastern countries. These poor prognostic factors explain the lower overall survival rate in Western randomized trials [2].

The incidence of gastric cancer increases with age, with 70 years being the median age at diagnosis. About 10% of cases are diagnosed before the age of 45 [3].

Gastric cancer is caused in approximatively 90% of cases by Helicobacter pylori infection. Its incidence is currently decreasing thanks to *Helicobacter pylori* eradication [4]; however, recent epidemiological studies show that its incidence is increasing in young patients which may be due to autoimmunity [5]. Early detection and follow-up of high-risk patients should help to define personalized treatment strategies [6].

Its incidence is highest in East and Central Asia and then in Latin America and Eastern Europe. The risk factors for gastric cancer include non-modifiable factors such as age and sex, but also modifiable factors, the most important of which is infection by Helicobacter pylori, and a diet that contains a high level of nitrates and nitrites. Rarer risk factors for gastric cancer include a history of mucosa-associated lymphoid tissue, Biermer’s disease, and a previous partial gastrectomy. In addition, there are familial cancers, in particular the hereditary diffuse gastric cancer CDH1 mutation, which leads to an 80% risk of developing gastric cancer especially touching young people and more rarely developing Lynch syndrome, breast and ovarian cancer BRCA, Li-Fraumeni, familial adenomatous polyposis and Peutz–Jegher syndromes.

A recent study showed that patients with a high genetic risk of developing gastric adenocarcinoma could decrease their risk by adopting a healthy lifestyle [7].

## 3. TCGA Classification towards Personalized Medicine

The classification of gastric cancers by molecular subtypes according to the TCGA classification [8] has potential for individualized therapy but has no routine application at this time.

The classification of gastric cancers based on molecular subtypes is a first step towards personalized medicine. Biomarkers such as microsatellite instability (MSI), programmed death ligand 1 (PD-L1), human epidermal growth factor 2 (HER2), and Epstein–Barr virus, are increasingly used routinely to identify patients who may benefit from targeted therapies but currently constitute a small proportion of gastric cancer patients. Progress is needed for poorly differentiated subtypes or in the absence of biomarkers [9].

## 4. Diagnosis, Pre-Therapeutic Assessment and Staging

The diagnosis of gastric and esophageal cancer is based on upper gastrointestinal endoscopy with biopsies taken in the presence of symptoms including dyspepsia and reflux but also in signs of advanced disease such as hematemesis, weight loss or dysphagia. However, a precise description of the location and extension of the primary tumor is often lacking and it is often necessary to repeat the endoscopy associated with an echo-endoscopy to optimize the staging. Echo-endoscopy is very useful to evaluate the possibility of performing an endoscopic resection for T1 tumors. The extension workup is completed using chest/abdomen/pelvic computed tomography which can be completed with a fluorodeoxyglucose-positron emission tomography/CT scan (PET scan) in specific situations for the evaluation of indeterminate lesions. However, the PET-scan is often not very informative for poorly differentiated tumors, signet ring cells type histology or mucinous tumors.

Exploratory laparoscopy facilitates the identification of peritoneal carcinosis in about 20% of patients without radiological images of metastasis [10].

Appropriate initial staging is essential for the prompt initiation of systemic therapy.

In particular, the search for microsatellite instability is of interest at the time of diagnosis because adverse oncologic outcomes after standard oncologic treatment have been demonstrated in MSI patients [11]. The place of immunotherapy in these patients deserves to be better defined in the future in light of the results of randomized trials [12].

## 5. Multidisciplinary Treatment

### 5.1. Perioperative Chemotherapy

Non-metastatic gastric cancer from T2N0 requires multidisciplinary management including perioperative chemotherapy. The MAGIC trial demonstrated a survival benefit in patients who received perioperative chemotherapy such as epirubicin, cisplatin, and fluorouracil (ECF) versus surgery alone (5-year overall survival 36 vs. 23%) [13].

More recently, the phase 2/3 FLOT4-AIO trial compared chemotherapy with FLOT (fluorouracil plus leucovorin, oxaliplatin and docetaxel) versus ECF in patients with resectable esophageal adenocarcinoma and demonstrated an improvement in 5-year overall survival in the FLOT group (45 vs. 36%) [14]. The FLOT regimen has consequently become the new standard of treatment has perioperative chemotherapy for non metastatic gastric cancer. Research on the value of adding targeted therapy in patients expressing certain biomarkers in addition to FLOT is currently very active.

Patients who have had primary surgery, T3, T4 or N+ should receive adjuvant chemotherapy [9].

### 5.2. Endoscopic Resection

Gastric cancer is more rarely diagnosed at an early stage in Western countries than in Eastern countries. The current criteria for endoscopic resection are the absence of deep submucosal invasion, lymphovascular invasion and a diameter of less than 2 cm.

### 5.3. Surgical Treatment

Surgical approaches for gastric cancer are total or subtotal gastrectomy combined with D2 lymphadenectomy due to the lymphophilic nature and propensity for cancer cells to spread in the gastric wall to facilitate resection with negative resection margins [9].

The extension of lymphadenectomy is strongly debated in the literature concerning tumors of the esogastric junction. Concerning esogastric junction tumors, a recent metanalysis studied the incidence of lymph node metastasis by defining mapping useful for lymphadenectomy extension in Siewert II and III tumors [15].

In addition, minimally invasive surgery is increasingly discussed in the literature but is a challenging procedure. A recent meta-analysis demonstrated the benefits of the robotic approach compared to the laparoscopic approach showing a decrease in complications with similar oncological results [16].

### 5.4. Treatment of Unresectable and Metastatic Gastric Cancer

Several chemotherapies are effective in patients with advanced gastric cancer including irinotecan, taxanes, platinum and fluoropyrimidines. The decision to treat depends on the patient’s performance status, comorbidities and the toxicities of previous treatments. A combination of drugs provides a higher response rate and improves survival compared to single-agent therapy.

This is a palliative treatment, the goal of which is to control the disease and prolong life. The first-line therapy is typically a combination of fluoropyrimidine and platinum, in particular oxaliplatin or cisplatin [17]. In patients in very good general condition, triple chemotherapy combining fluoropyrimidin, oxaliplatin and docetaxel can be proposed, but there is no consensus on the best approach after first-line failure [18,19].

In patients with HER2 overexpression, trastuzumab should be added to the first line of chemotherapy. Furthermore, for patients with a programmed death ligand 1 (PD-L1) combined positive score (CPS) ≥ 5, nivolumab should be added. In second-line treatment in a phase III trial for metastatic gastric cancer, cytotoxic chemotherapy not previously used in a patient with the possible addition of ramucirumab (a VEGFR2-binding monoclonal antibody) indicated a second-line survival gain of 1.4 months [20].

## 6. Targeted Theapies

### 6.1. Immunotherapy

In the last decade, immunotherapy in the management of cancer has become central in the therapeutic armamentarium.

#### 6.1.1. High Microsatellite Instability (MSI-H)/Mismatch Repair Deficient Tumor

The cancer genome atlas research network performed molecular characterization of 295 gastric adenocarcinomas and described the following four subtypes of gastric cancer: MSI, EBV, chromosomal instability and genomically stable. In this study, 22% of patients were found to have MSI-H [8].

It was shown that MSI-H tumors are resistant to chemotherapy and have a good response to immunotherapy [21].

#### 6.1.2. Tumor Mutations

However, gastric cancer has a variable response to immunotherapy especially due to tumor mutations and is a consequently a heterogeneous group of tumors.

### 6.2. Human Epidermal Growth Factor 2 (HER2)-Positive Gastric Cancer

HER2-positive gastric cancer accounts only for 15–20% of gastric and esophageal cancers. In these cases, Trastuzumab plus chemotherapy improves overall survival compared to chemotherapy alone in patients with advanced gastric cancer [22].

## 7. Prognosis

Currently, the prognosis for stage IA or IB tumors can reach a 5-year survival rate of 60–80%. However, stage III tumors that are operated on can have a survival rate of 18–50% depending on the treatment strategy. This highlights the need for a more effective treatment strategy guided by molecular data [23].

## 8. Perspectives

Advances in cancer biology and sequencing have facilitated the selection of targeted therapies and the selection of more effective treatments. Currently, only the following three biomarkers are routinely used in gastric cancer: HER2, PD-L1 and MSI. Despite a lack of clinically relevant biomarkers, many clinical trials are underway to study the expression of biomarkers that could provide insights into intratumoral heterogeneity conditioning the response to treatment. This could lead to the improved selection of patients to receive targeted therapies [24].

Moreover, drug resistance is a very important field of research in the management of gastric cancer because it leads to treatment failure and a poor outcome. Several mechanisms of drug resistance have been highlighted, including non-coding RNAs such as microRNAs (miRNAs), long non-coding RNAs (lncRNAs) circular RNAs (circRNAs) [25], and small extracellular vesicles [26].

In addition, to improve the outcome of gastric cancer treatment, new targets from epithelial–mesenchymal transition (EMT) and cell–cell adhesion are being studied. Indeed, EMT plays a role in metastasis initiated by the loss of cell–cell adhesion. Claudins are highly expressed in gastric cancer and are a potential therapeutic target currently under investigation [27].

Recently gastric cancer stem cells have been identified as playing an important role in the initiation and progression of gastric cancer, have been characterized as metastasis-initiating cells and represent a prime target of current research [28].

## 9. Conclusions

Thus, there are still research opportunities for the less differentiated histological types of gastric adenocarcinoma and those that do not overexpress the biomarkers used in current practices.
